# Colorful Treasure From Agro-Industrial Wastes: A Sustainable Chassis for Microbial Pigment Production

**DOI:** 10.3389/fmicb.2022.832918

**Published:** 2022-01-31

**Authors:** Jasneet Grewal, Mikołaj Woła̧cewicz, Weronika Pyter, Namrata Joshi, Lukasz Drewniak, Kumar Pranaw

**Affiliations:** Department of Environmental Microbiology and Biotechnology, Institute of Microbiology, Faculty of Biology, University of Warsaw, Warsaw, Poland

**Keywords:** agro-industrial wastes, microbial pigments, natural colorants, engineered microbes, fermentation, biological activities

## Abstract

Colors with their attractive appeal have been an integral part of human lives and the easy cascade of chemical catalysis enables fast, bulk production of these synthetic colorants with low costs. However, the resulting hazardous impacts on the environment and human health has stimulated an interest in natural pigments as a safe and ecologically clean alternative. Amidst sources of natural producers, the microbes with their diversity, ease of all-season production and peculiar bioactivities are attractive entities for industrial production of these marketable natural colorants. Further, in line with circular bioeconomy and environmentally clean technologies, the use of agro-industrial wastes as feedstocks for carrying out the microbial transformations paves way for sustainable and cost-effective production of these valuable secondary metabolites with simultaneous waste management. The present review aims to comprehensively cover the current green workflow of microbial colorant production by encompassing the potency of waste feedstocks and fermentation technologies. The commercially important pigments viz. astaxanthin, prodigiosin, canthaxanthin, lycopene, and β-carotene produced by native and engineered bacterial, fungal, or yeast strains have been elaborately discussed with their versatile applications in food, pharmaceuticals, textiles, cosmetics, etc. The limitations and their economic viability to meet the future market demands have been envisaged. The most recent advances in various molecular approaches to develop engineered microbiological systems for enhanced pigment production have been included to provide new perspectives to this burgeoning field of research.

## Graphical Abstract

An illustration exhibiting perusal of natural pigment production as a safe alternative to synthetic ones.

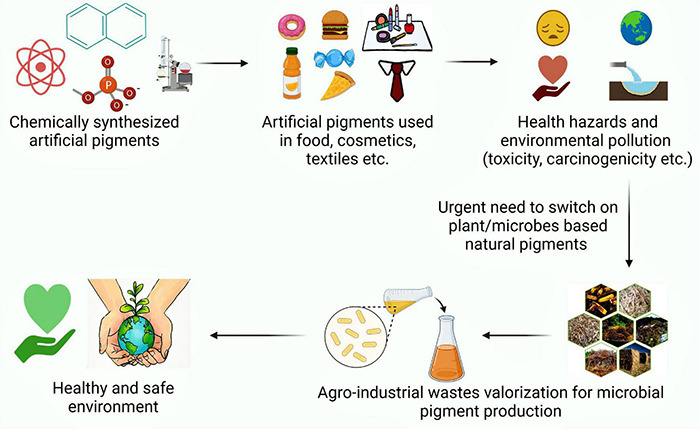

## Introduction

Color has been a part of human lives for centuries and has been an integral component to increase the desirability of any product. Apart from multiple cultural meanings, color has enormous market potential in food, agriculture, cosmetic, textile, pharmaceutical, and nutraceutical sectors. The molecules capable of bestowing color, owing to their ability to absorb light in the visible range are referred as pigments. Nevertheless, by chemical definitions, pigments are insoluble colored substances while colorants are soluble colored substances (Rana et al., 2021). By 2027, the market value of dyes and pigments is expected to reach 33.2–49.1 billion dollars (Chatragadda and Dufossé, 2021). Though synthetic petro-derived colorants have dominated the industries due to low cost and high yield, the rising concerns about their non-biodegradability, carcinogenicity, and environmental toxicity have enthused both industry and researchers to find natural and safe alternatives. In this context, natural and eco-friendly biopigments have sparked great interest due to their non-toxicity, biodegradability, non-carcinogenicity, and non-allergenicity which increases their consumer acceptance, prevents occupational health hazards while negating environmental concerns (Pailliè-Jiménez et al., 2020; Aman Mohammadi et al., 2021; Mussagy et al., 2021).

Among natural sources, microbial pigments are an attractive target as compared to plant or animal sources due to their fast growth, all-season availability and ease of regulating microbial cell factories for high production yields. Therefore, microbial pigment production by fermentative technology is a dynamic toolkit to produce a plethora of stable and safe pigments. Nonetheless, economic and marketing difficulties remain the key concern for microbial production at a commercial scale (Ramesh et al., 2019; Aman Mohammadi et al., 2021). The microbial growth medium is one of the vital parameters contributing to fermentative production cost. Therefore, the use of inexpensive waste substrates as a growth medium can translate to the low cost of pigment production. In this context, the agro-industrial residues have the potential to serve as ideal substrates for microbial pigment production. These residues left unutilized add to waste generation which is expected to reach 3.40 billion metric tons by 2050 (Usmani et al., 2020; Sodhi et al., 2021). The unregulated dumping of waste has already made waste management a humongous challenge, which requires serious addressal to prevent environmental and health hazards. The conventional methods of waste management *via* landfilling, open dumping, burning, or incineration of waste have been ridiculed as they lead to an increase in carbon footprint. Henceforth, the focus has shifted on waste valorization which provides the dual benefit of sustainable production of value-added products and waste management. Conventionally, most of the technological advancements focused on the generation of biofuels or bioenergy from these waste feedstocks (Jatoi et al., 2021). However, recently, they have emerged as potent substrates for the production of a myriad of value-added products viz. enzymes, platform chemicals, bioactive molecules. etc. (Grewal and Khare, 2017, 2018; Grewal et al., 2020; Lu et al., 2022). On similar lines, these agro-industrial residues have been found to be suitable as nutrient supports for the growth of microorganisms and produce pigments by both submerged and solid-state fermentation (SSF). This approach not only combats environmental problems but also provides a sustainable and cost-effective framework for developing the circular bioeconomy (Banu et al., 2021; Muscat et al., 2021; Yaashikaa et al., 2022).

Since a majority of the agro-industrial residues are lignocellulosic in nature, their recalcitrance necessitates pretreatment of these residues before microbial conversion. Various pretreatment approaches viz. physical, chemical, physico-chemical, or biological have been applied for different lignocellulosics with each offering its own advantages and constraints (Ali et al., 2020; Haldar and Purkait, 2020; Mankar et al., 2021). These pretreatment processes aid in the structural deconstruction of complex polymeric linkages, allowing improved accessibility to hydrolytic enzymes during saccharification (Grewal et al., 2017; Zhang H. et al., 2021). For further microbial bioprocessing of these untreated or pretreated residues for pigment production, both submerged and SSF techniques have been effectively used (Aman Mohammadi et al., 2021; Rana et al., 2021; Sodhi et al., 2021).

The present work comprehensively encompasses utilization of various agro-industrial residues for pigment production. The production of pigments from various native microbial sources viz. bacteria, fungi, and yeast has been elaborately discussed along with an emphasis on fermentation technologies. Further, the recent perspectives on different molecular, genetic, or metabolic approaches for creating engineered strains with a high titer of stable pigment production have been appraised. The versatile applications of microbial pigments are also encompassed. Overall, the illustration of all these diverse aspects will help to contribute to the development of cost-effective bioprocesses for the production of natural colorants from waste feedstocks with positive societal, industrial, and environmental implications.

## Major Classes of Pigments and Microbial Sources

In general, the natural pigments can be majorly classified into the following categories based on their structural characteristics: (i) melanins; (ii) quinones: anthraquinone, naphthoquinone, and benzoquinone; (iii) benzopyran derivatives: flavonoids and anthocyanins; (iv) isoprenoid derivatives: iridoids and carotenoids; (v) tetrapyrrole derivatives: heme and chlorophylls; and (vi) other N-heterocyclic compounds: betalains, phenazines, flavins, phenoxazines, purines, and pterins (Delgado-Vargas et al., 2000). The different classes of microbes, i.e., bacteria, fungi, yeast, and algae produce a diverse array of pigments viz. carotenoids, flavins, anthraquinones, violacein, and prodigiosin, of which most prominent ones are discussed below.

Carotenoids, one of the most diverse class of pigments belongs to a subfamily of isoprenoids and is constituted by eight isoprene units. They are further categorized into carotenes and xanthophylls. Carotenes are constituted by carbon and hydrogen whereas the xanthophylls or oxycarotenoids contain carbon, hydrogen, and oxygen (Delgado-Vargas et al., 2000; Gmoser et al., 2017). Amidst these carotenoids, capxanthin, lutein, astaxanthin, canthaxanthin, lycopene, and β-carotene have very high market demand (Gmoser et al., 2017; Ramesh et al., 2019). The global market of carotenoids growing at a compound annual growth rate (CAGR) of 2.6% is expected to reach 2 billion dollars by 2027 (Mussagy et al., 2021). However, the chemical synthesis constitutes 80–90% of carotenoids supply for the market and hence, to counter their side-effects, the microbial sources are gaining high demand (Saini and Keum, 2019; Chatragadda and Dufossé, 2021). Though some companies such as Cyanotech (United States), Algatech (Israel), and Parry Nutraceuticals (India) have started producing carotenoids by biotechnological route, their market share is substantially smaller than synthetic producers (Mussagy et al., 2021). The pathway of carotenoid biosynthesis and its regulation has been the subject of immense interest across diverse producers and is among the critical and popular targets for modulating pigment production (Delgado-Vargas et al., 2000; Usmani et al., 2020).

Lycopene, identified as a class A nutrient by WHO (World Health Organization) and FAO (United Nations Food and Agriculture Organization) is an unsaturated lipophilic isoprenoid pigment with multiple physiological functions and myriad applications especially in preventive health care (Li L. et al., 2020). The global lycopene market, expanding at a CAGR of 5.0% is expected to reach 161 million dollars in 2025 from the net worth of 126 million dollars estimated in 2020 (Mussagy et al., 2021). Anthocyanins are another class of important glycosylated pigments responsible for different attractive colors and majorly produced by plants as secondary metabolites. Its market value is expected to reach 228.4 million dollars in 2027 from the net worth of 192.5 million dollars estimated in 2020 (Mussagy et al., 2021). Nonetheless, the engineering of the anthocyanin biosynthetic pathway is under intensive research to produce this pigment from genetically modified microbial sources (Zha and Koffas, 2017; Usmani et al., 2020).

### Bacterial Pigments

Many of the bacteria appear highly attractive due to the color they exhibit viz. *Gordonia jacobaea*, *Serratia marcescens (red)*; *Chromobacterium* sp. (purple); *Erwinia chrysanthemi*, *Corynebacterium insidiosum*, *Vogesella indigofera* (blue); *Chryseobacterium* sp., *Hymenobacter* sp., *Micrococcus* (yellow); *Chryseobacterium artocarpi*, *Kocuria* sp. (red-yellow), *Pseudomonas* sp. (green) (Venil et al., 2020a). Carotenoids, prodigiosin, tambjamines, melanins, quinones, and violacein are the most commonly produced bacterial pigments. These have been reported to exhibit antioxidant, antimalarial, and anticarcinogenic properties which make them suitable for food or biomedical applications (Pailliè-Jiménez et al., 2020; Sajjad et al., 2020). For carotenoids, the widest distributed class of biopigments with high commercial value, the genus *Dietzia and Paracoccus* are major producers (Gharibzahedi et al., 2014; Maj et al., 2020). It has been well established that the crt gene cluster is necessary for *Paracoccus* strains to produce carotenoids. Their pigment biosynthesis pathway and respective gene functions have been examined and characterized by several studies (Ide et al., 2012; Honda et al., 2020; Maj et al., 2020). Apart from these, *Micrococcus roseus*, *Corynebacterium michiganense*, *Bradyrhizobium* sp., *Brevibacterium* sp., *Agrobacterium* sp., *Streptomyces* sp., and *G. jacobaea* are among the popularly reported carotenoids producers (Ram et al., 2020; Venil et al., 2020a).

### Fungal and Yeast Pigments

Some filamentous fungi exhibit a wide spectrum of colors viz. red pigments by *Talaromyces* sp., deep blood red by *Cordyceps unilateralis*, orange by *Herpotrichia rhodosticta*, etc. The fungi belonging to families of *Chlorociboriaceae, Monascaceae, Sordariaceae, Trichocomaceae, Chaetomiaceae, Nectriaceae, Xylariaceae, Hypocreaceae, Cordycipitaceae, Pleosporaceae*, etc., are reported as prominent pigment producers (Ramesh et al., 2019; Pailliè-Jiménez et al., 2020). However, many pigment-producing fungi viz. *Penicillium* sp., *Aspergillus* sp., and *Fusarium* sp. also secrete secondary toxic metabolites or mycotoxins, which in turn becomes a safety constraint for their commercial applications. However, these bottlenecks are affected by the type of microbial strain, the flux of carbon oriented towards the correct pigment pathway as well as country regulations. For example, though *Monascus* sp. is popularly used for the production of fermented food products in Asian countries for 1,000 years, their use as food colorants is not approved in the United States or European Union due to the co-production of citrinin, exhibiting hepatotoxic, nephrotoxic, or carcinogenic properties (Gmoser et al., 2017; He et al., 2021). Nevertheless, the major azaphilone pigments produced by *Monascus* sp. are of immense interest for varied applications. These include yellow pigment (ankaflavin and monascin), red pigment (rubropunctamine and monascorubramine), and orange pigment (monascorubrin and rubropunctatin) (Dufossé, 2018b; He et al., 2021). Similarly, *Blakeslea trispora* is another fungal sp. that has been of immense interest for lycopene production and has been intensively investigated by both researchers and industries. This strain in both native and engineered forms has exhibited great potential for high titer lycopene production (Li L. et al., 2020). Another mold that has been widely reported for β-carotene production at an industrial scale is *Phycomyces blakesleeanus* (Mata-Gómez et al., 2014). The filamentous fungi such as *Neurospora* sp. are also being investigated for harnessing their pigment potential due to their GRAS (generally recognized as safe) status and non-mycotoxigenic nature (Gmoser et al., 2017; Meruvu and Dos Santos, 2021).

The specific genera of yeast viz. *Pichia, Rhodotorula, Xanthophyllomyces, Rhodosporidium, Sporobolomyces*, and *Sporidiobolus* are also potent producers of various carotenoids and other pigments. The most commonly produced carotenoids by yeast include β-carotene, torulene, astaxanthin, and canthaxanthin (Mata-Gómez et al., 2014; Rapoport et al., 2021). For the optically active astaxanthin, the 3R, 3′R isomeric form is produced by *Xanthophyllomyces dendrorhous*. The torularhodin produced by *Sporobolomyces* or *Rhodotorula* genera has robust antimicrobial properties which makes it suitable for many pharmaceutical applications (Rapoport et al., 2021).

## Agro-Industrial Wastes as Low-Cost Substrates for Pigment Production

**FIGURE 1:**
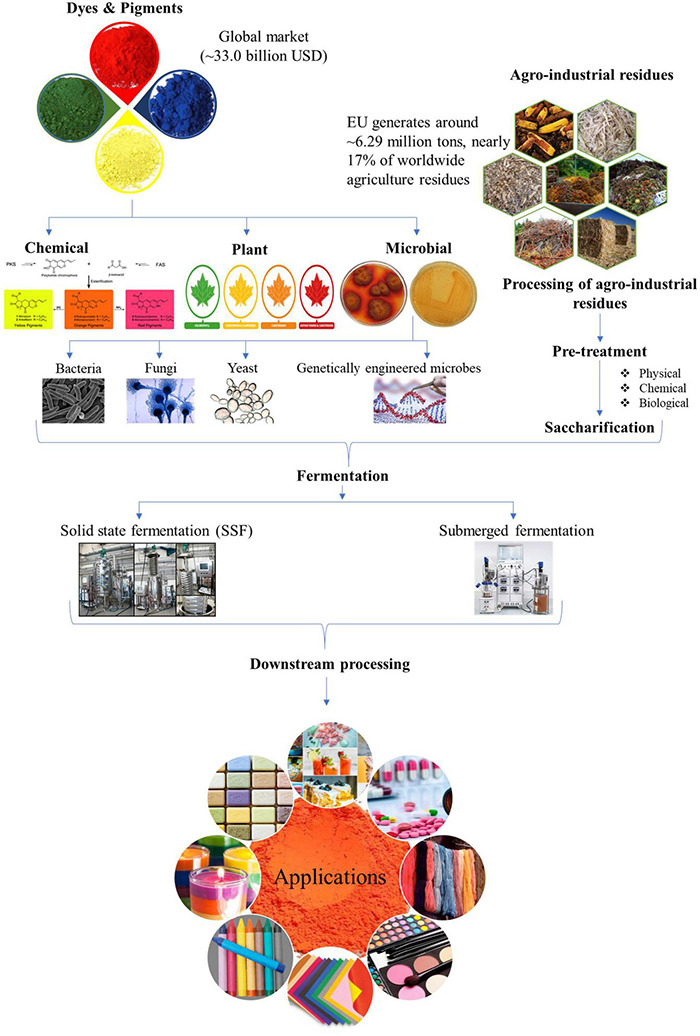
Schematic representation for microbial pigment production by valorization of agro-industrial wastes.

The high production cost for microbe derived pigments is a severe deterrent, especially at pilot or industrial scale, to compete with synthetic pigments as safe alternatives. The expensive synthetic medium used for culturing microbes for pigment production is one of the critical factors affecting the cost of biotechnological synthesis. In this perspective, the agro-industrial wastes available in abundance have immense potential to be harbored as low-cost substrates for decreasing the production cost (Venil et al., 2017; Usmani et al., 2020). The processing of agricultural crops, post-harvesting operations and by-products of industrial processes generate enormous amounts of residues which if left untreated act as pollutants to the environment. The valorization of these wastes by utilizing them as feedstocks for pigment production helps in combating environmental and health hazards while simultaneously adding value to the development of economical bioprocess. Nonetheless, their effective utilization will depend on the nature of their raw composition and will influence the processing steps. For example, lignocellulosic wastes such as corncob, sugarcane bagasse, rice straw, wheat straw, and rice husk will require the deployment of an optimal and economical pretreatment approach before they can be used for hydrolyzate production by the action of saccharifying enzymes (Sodhi et al., 2021; Zhang H. et al., 2021). Apart from biomass-derived hydrolyzates used as a nutrient source for pigment production *via* submerged fermentation, the use of waste substrates as matrix *via* SSF has also been widely reported (Gmoser et al., 2019; Chen et al., 2021; Dos Santos et al., 2021; Kumar Shetty et al., 2021). The schematic representation of valorization of agro-wastes for pigment production is denoted in Figure 1.

The effective usage of various waste substrates for pigment production will depend on the availability of waste, its raw composition and the nutrient requirement of fermenting microorganisms. The ability of *Talaromyces atroroseus* GH2 to co-utilize glucose and xylose enabled economically competitive pigment production (16.17 ± 0.37 OD_500*nm*_) from acid hydrolyzate of corncob without nutrient supplementation (Morales-Oyervides et al., 2020b). The detoxification of lignocellulosic hydrolyzates used as a nutrient source, is another processing step that could be required to improve the titer of pigment production. The study employing loquat (*Eriobotrya japonica*) kernels as a substrate for carotenoids production by *Rhodotorula glutinis* MT-5 reported 72.36 and 62.73 mg/L of carotenoids from the fermentation of detoxified and non-detoxified loquat kernel extract respectively (Taskin and Erdal, 2011). Similarly, the Ca(OH)_2_ mediated detoxification of brewer’s spent grain (BSG) hydrolyzate enabled its utilization by *Monascus purpureus* CMU001 to give a red pigment yield of 22.25 UA_500_ (Silbir and Goksungur, 2019). In another approach, a mutant strain of *M. purpureus* M523 was generated by the use of atmospheric and room temperature plasma (ARTP) screening system, which exhibited high tolerance to non-detoxified acid hydrolyzate of rice husk. The employment of this mutant strain spores immobilized in sodium alginate, enabled pigment production of 80.7 U/mL from rice husk (Zhang S. et al., 2021).

The problem of toxicity of simultaneous mycotoxin production by many pigment-producing fungi was effectively countered in the work of Gmoser et al. (2019) as the edible filamentous fungi *Neurospora intermedia* was shown to produce 1.2 kg pigment per ton of waste bread valorized. Similarly, the pigment (0.565 AU/mL) produced by *Talaromyces purpureogenus* CFRM02 after utilizing *Cicer arietinum* (Bengal gram) husk as a substrate for fermentation was proved to be non-toxic making it potentially applicable as a natural colorant for food or other nutraceutical applications (Pandit et al., 2019).

In another interesting study (Hilares et al., 2018), employing lignocellulosic hydrolyzate, red pigment production by *Monascus ruber* Tieghem IOC 2225 was 2.5 times higher using sugarcane bagasse hydrolyzate as compared to glucose-based medium under similar conditions. Thus, it was inferred that the complexity of the medium could influence the metabolic pathway and consequently, affect the titer and diversity of secondary metabolites produced by the fermenting microbe. In another approach, the cells of *Chromobacterium violaceum* were immobilized on sugarcane bagasse to form a continuous column system which resulted in the production of 0.15 g/L of violacein (Venil et al., 2017). The use of glycerol, a by-product of biodiesel manufacture as a carbon source by *R. glutinis* TISTR 5159 led to concomitant production of lipids (6.05 g/L) as well as carotenoids (135.25 mg/L) in a stirred tank bioreactor (Saenge et al., 2011). Among the pioneer reports on biopigment production by *Streptomyces* genus utilizing agro-industrial wastes, Schalchli et al. (2021) reported 1.75 mg/g pigment production by *Streptomyces* sp. strain SO6 utilizing potato waste as the sole nutrient source. Recently, cost-effective scaled-up production of prodigiosin (6886 mg/L) by *S. marcescens* TUN02 was achieved in a 14-L bioreactor system by using groundnut cake as the sole carbon/nitrogen source. The anti-nematode activity exhibited by purified prodigiosin exhibited potential for agricultural applications (Nguyen et al., 2022). Table 1 summarizes the various agro-industrial wastes used for pigment production by various microbes *via* both submerged as well as SSF.

**TABLE 1:** Microbial pigment production by utilizing various agro-industrial wastes as substrates.

Agro-industrial wastes	Microorganism	Type of fermentation process	Microbial pigment	Titer	References
Groundnut cake	*Serratia marcescens* TUN02	Submerged	Prodigiosin (red)	6,886 mg/L	Nguyen et al., 2022
Whey	*Monascus purpureus*	Submerged	*Monascus* pigments (red)	38.4 UA_510*nm*_ (absorbance units)	Mehri et al., 2021
Rice husk	*Monascus purpureus* M523	Submerged	*Monascus* pigments (yellow, orange)	80.7 U/mL	Zhang S. et al., 2021
Waste soybean oil, wheat bran	*Serratia marcescens* UCP 1549	SSF (solid-state fermentation)	Prodigiosin (red)	119.8 g/kg	Dos Santos et al., 2021
Potato pomace	*Monascus purpureus* CH01	SSF	*Monascus* pigments (red, yellow)	1,922.7 OD unit/g	Chen et al., 2021
Broken rice	*Monascus sanguineus* NFCCI 2453	SSF	*Monascus* pigments (red)	143.3 OD U/gds	Kumar Shetty et al., 2021
Potato waste	*Streptomyces* spp.	Submerged	Reddish-purple biopigment	1.75 mg/g	Schalchli et al., 2021
Loquat (*Eriobotrya japonica* L.) kernels	*Monascus purpureus* ATCC16365	Submerged	*Monascus* pigments (red, orange, and yellow)	327, 241, and 204 AU/L, respectively	Arslan et al., 2021
Shrimp head powder (fish processing waste)	*Serratia marcescens* CC17	Submerged	Prodigiosin (red)	6,310 mg/L	Nguyen et al., 2021
Wheat bran	*Chromobacterium vaccinii*	Submerged	Violacein (violet)	1.47 mg/L	Cassarini et al., 2021
Rice straw hydrolyzate	*Monascus purpureus* M630	Submerged	*Monascus* pigments (ankaflavin and monascin; yellow, monascorubrin and rubropunctatin; orange, monascorubramine and rubropunctamine; red)	8.61 U/mL	Liu et al., 2020b
Corncob hydrolyzate	*Talaromyces atroroseus* GH2	Submerged	Monascorubrin, cribrarione, monaphilone, *N*-threoninerubropunctamine (bright and dark red)	16.17 ± 0.37 OD_500*nm*_	Morales-Oyervides et al., 2020b
Waste bread	*Neurospora intermedia*	SSF	Carotenoids (yellow/orange to reddish)	1.2 kg/ton bread	Gmoser et al., 2019
Bengal gram (*Cicer arietinum*) husk	*Talaromyces purpureogenus* CFRM02	Submerged	Peniazaphilone-A, PP-R (red)	0.565 ± 0.05 AU/mL	Pandit et al., 2019
Brewer’s spent grain (BSG)	*Monascus purpureus* CMU001	Submerged	Rubropunctamine and monascorubramine (natural red)	22.25 UA_500*nm*_	Silbir and Goksungur, 2019
Sugarcane bagasse hydrolyzate	*Monascus ruber* Tieghem IOC 2225	Submerged	*Monascus* pigments (red)	18.71 AU_490*nm*_	Hilares et al., 2018
Waste orange peels	*Monascus purpureus* ATCC 16365	SSF, submerged	*Monascus* pigments (yellow, red, orange)	9 AU/gds in SSF 0.58 AU/mL in submerged	Kantifedaki et al., 2018
Sugarcane bagasse	*Chromobacterium violaceum*	Submerged	Violacein (violet)	0.822 g/L	Venil et al., 2017
Cotton seed meal	*Pseudomonas aeruginosa* R1	Submerged	Pyocyanin (blue green)	4.0 μg/mL	El-Fouly et al., 2015
Liquid pineapple waste	*Chromobacterium violaceum* UTM5	Submerged	Violacein (violet)	16,256 +440 mg/L	Aruldass et al., 2015
Cheese whey, grape waste	*Penicillium chrysogenum* IFL1	Submerged	Chrysogenin (yellow)	74.7 AU/mL on cheese whey 46.50 AU/mL on grape waste	Lopes et al., 2013
Corncob hydrolyzate	*Penicillium resticulosum* Blr1	Submerged	Red pigment	497.03 +55.13 mg/L	Sopandi et al., 2013
Whey waste	*Rhodotorula glutinis* CCY 20-2-26	Submerged	β-Carotene (red-orange)	46 mg/L	Marova et al., 2012
Crude glycerol from biodiesel plants	*Rhodotorula glutinis* TISTR 5159	Submerged	Carotenoids (red)	135.25 mg/L	Saenge et al., 2011
Loquat (*Eriobotrya japonica* Lindl.) kernels	*Rhodotorula glutinis* MT-5	Submerged	Carotenoids (orange, red)	62.73 mg/L	Taskin and Erdal, 2011
Corn meal, coconut residue, peanut meal, soybean meal	*Monascus purpureus* CMU001	SSF	*Monascus* pigments (red)	129.63 U/gds corn meal, 63.50 U/gds coconut residue, 52.50 U/gds peanut meal, 22.50 U/gds soybean meal	Nimnoi and Lumyong, 2011
Sugarcane waste	*Streptomyces* sp.	Submerged	Melanin (brown, black)	21.13 g/l	Vasanthabharathi et al., 2011
Fermented radish brine	*Rhodotorula glutinis* DM28	Submerged	β-Carotene (orange-yellow)	201 μg/l	Malisorn and Suntornsuk, 2008

### The Economic Viability of Pigment Production From Valorization of Agro-Industrial Residues

In a promising study by Dursun et al. (2020), a detailed techno-economic analysis for industrial bioprocess of astaxanthin production from wheat bran by *X. dendrorhous* using SSF was carried out. The results depicted a $3.9 million gross profit for astaxanthin production, strengthening the agro-industrial waste valorization as a feasible technology for pigment production. Similarly, the cost for violacein production by *C. violaceum* UTM5 was decreased to 235.70 USD by using liquid pineapple waste as compared to 281.20 USD in nutrient broth (Aruldass et al., 2015). The produced pigment also exhibited good stability and anti-microbial activity against two strains of *Staphylococcus aureus*, one of which was methicillin-resistant. In another study (Saejung and Puensungnern, 2020) supporting the economic viability of using wastes, the use of molasses-based medium for carotenoid production by *Rhodopseudomonas faecalis* PA2 reduced cost by 90.88% as compared to synthetic chemical medium despite exhibiting similar carotenoid productivity in both media. Moreira et al. (2018) used yeast strain-*Rhodotorula mucilaginosa* CCMA 0156 to ferment coffee waste and reported a production cost of $5.04/g β-carotene while the commercial value of synthetic β-carotene was around $10.40/g. Further, the exhibited antimicrobial and antioxidant properties of the obtained pigment added value to this natural product produced at lower costs. Recently, the study (Mehri et al., 2021) employing demineralized whey, a dairy industry waste, for red pigment production by *M. purpureus* reported equivalent operational cost (∼14.92 dollars/kg) in comparison to using glucose as the equivalent nutrient source. These studies suggest it is imperative to evaluate the economic feasibility of waste derived fermentation processes. However, still there are no extensive research studies available to critically evaluate the techno-economic and life-cycle assessment (LCA) aspects of waste derived biorefineries to sustain the viability of pigment production at industrial scale. Nonetheless, the evaluation of economic viability coupled with social and political implications of altering the health and environmental hazards by sustainable technologies will pave a way for production of natural pigments.

Overall, though utilization of by-products is a sustainable strategy for minimizing environmental contamination and concomitant pigment production, it has major bottlenecks. One of the major challenges encountered is the limited potential of natural pigment producers, which have been majorly used for production from agro-industrial wastes. Therefore, the use of genetic, metabolic or protein engineering could be a viable strategy for developing improved strains for pigment production, which has been elaborated in further sections.

## Genetic and Metabolic Engineering Approaches for Enhanced Pigment Production by Strain Improvement

Microorganisms generally are considered conducive for pigment production due to the ease, safety and short span of the fermentation cycle without being limited by geographical or seasonal influence. Nonetheless, the development of molecular biology has made it possible to manipulate the genome of microorganisms to such an extent that we can obtain new features as well as upregulate the selective expression of genes to get high yields of the desired product. In this way, efficient microbiological systems can be created for the valorization of biomass with the simultaneous production of value-added products such as pigments (Usmani et al., 2020; Wan et al., 2020; Wang et al., 2021). Table 2 summarizes the engineered microbes for pigment production using different genomic or metabolic strategies.

**TABLE 2:** Engineered microbes for pigment production using various strain improvement strategies.

Microorganism	Engineering strategy	Pigment	Titer	References
*Escherichia coli*	Carotenoid-pathway engineering and overexpression of capsanthin/capsorubin synthase	Capsanthin	0.5 mg/L	Furubayashi et al., 2021
	Combinatorial engineering based on Single Strand Assembly (SSA) methods and Golden Gate Assembly	Lycopene	448 mg/g CDW	Coussement et al., 2017
	Tricarboxylic acid cycle (TCA) and pentose phosphate pathway (PPP) modules engineering	β-Carotene	2.1 g/L	Zhao et al., 2013
	Multiplex Automated Genome Engineering (MAGE)	Lycopene	∼9,000 μg/g CDW	Wang et al., 2009
	Heterologous expression of mevalonic acid (MVA) pathway	β-Carotene	465 mg/L	Yoon et al., 2009
*Rhodobacter sphaeroides*	Multivariate Modular Metabolic Engineering (MMME)	Lycopene	10.32 mg/g CDW	Su et al., 2018
*Corynebacterium glutamicum*	Regulatory engineering involving targeted gene deletion and heterologous overexpression of specific genes of carotenoid pathway	Astaxanthin	10 mg/L	Henke et al., 2018
	Anthocyanidin synthase (ANS) and 3-O-glucosyltransferase (3GT) co-expression	Cyanidin 3-O-glucoside (an anthocyanin)	∼40 mg/L	Zha et al., 2018
*Paracoccus aminophilus* CRT1*; Paracoccus kondratievae* CRT2	pCRT01 plasmid expression	Carotenoids	452.5 μg/g CDW, 265.4 μg/g CDW, respectively	Maj et al., 2020
*Saccharomyces cerevisiae*	Lipid engineering and overexpression of key genes associated with fatty acid synthesis	Lycopene	73.3 mg/g CDW	Ma et al., 2019
	Heterologous module engineering and atmospheric and room temperature plasma (ARTP) mutagenesis	Astaxanthin	217.9 mg/L	Jin et al., 2018
	Modular assemblies of limiting enzymes (CrtE and IDI) in carotenoid biosynthetic pathway	Lycopene	2.3 g/L	Kang et al., 2019
	Heterologous expression of specific anthocyanin biosynthetic genes from *Arabidopsis thaliana* and *Gerbera hybrida*	Anthocyanidin pelargonidin	0.01 μmol/g CDW	Levisson et al., 2018
*Yarrowia lipolytica*	Carotenoid gene-promoter pair optimization and Golden Gate Assembly	β-Carotene	6.5 g/L	Larroude et al., 2018
*Monascus purpureus* HJ11	Activation of cAMP signaling pathway by knocking out cAMP phosphodiesterase gene (mrPDE)	*Monascus* azaphilones	8,739 U/g CDW	Liu et al., 2020a

The simplest and most frequently used technique is the use of expression plasmids to obtain higher expression of specific genes. This approach was effectively used by Maj et al. (2020) for the *in vivo* creation of plasmids which transformed colorless strains of *Paracoccus* sp. into efficient producers of xanthophylls and carotenes. Briefly, the crt gene cassette derived from the *Paracoccus marcusii* OS22 was used for creation of new plasmid pCRT01 by illegitimate recombination between the pABW1 vector carrying crt and the natural *paracoccal* plasmid pAMI2. This plasmid was transferred to two fast-growing but colorless *Paracoccus* strains by triparental mating (using *E. coli* DH5α strain as helper strain). The transformed strains, i.e., *Paracoccus aminophilus* CRT1 and *Paracoccus kondratievae* CRT2 could effectively grow on industrial effluents, i.e., flue gas desulfurization (FGD) wastewater supplemented with molasses and produced 127.09 and 58.80 ng/mL carotenoids, respectively. Using an analogous approach, Furubayashi et al. (2021) reported for the first time, the microbial production of capsanthin, a characteristic carotenoid found generally in *Capsicum annuum*. A heterologous capsanthin bio-synthetic pathway was engineered in *Escherichia coli by* expressing eight genes which included five zeaxanthin biosynthesis genes from a soil bacterium (*Pantoea ananatis*), zeaxanthin epoxidase (ZEP) and capsanthin/capsorubin synthase (CCS) from *C. annuum* and isopentenyl diphosphate isomerase (IDI) from green alga (*Haematococcus pluvialis*). After critical upregulation of carotenogenic genes and minimizing by-product formation, the production level of 0.5 mg/L capsanthin was attained.

Henke et al. (2016) engineered the carotenoid biosynthetic pathway for astaxanthin production by regulating the expression of heterologous genes encoding CrtY lycopene cyclase, CrtW β-carotene ketolase, and CrtZ hydroxylase in recombinant *Corynebacterium glutamicum.* The obtained volumetric productivity of 0.4 mg/L/h was competitive with the industrially used microalga *Dunaliella bardawil*. In their further study (Henke et al., 2018), an engineered *C. glutamicum* was developed for the combined overproduction of secreted l-lysine with the cell-associated carotenoids, β-carotene, lycopene, decaprenoxanthin, zeaxanthin, astaxanthin, and canthaxanthin. Further, the successful use of pentose sugars, i.e., arabinose and xylose as feedstock for co-production of l-lysine and β-carotene made this engineered strain a potential choice for valorizing mixed sugars generated from lignocellulosic wastes. The engineering of *C. glutamicum* was also attempted for anthocyanin production by co-expressing two genes, i.e., anthocyanidin synthase (ANS) and 3-O-glucosyltransferase (3GT) from *Petunia hybrida* and *Arabidopsis thaliana*, respectively. The controlled regulation of expressed anthocyanin pathway and optimized fermentation parameters resulted in the production of 40 mg/L cyanidin 3-O-glucoside (C3G) from catechin (Zha et al., 2018).

A new assembly scheme reported by Coussement et al. (2017) also helped in the optimization of the lycopene biosynthesis pathway in engineered *E. coli* to achieve 448 mg lycopene/g cell dry weight (CDW). The approach involved combining Single Strand Assembly (SSA) methods (Coussement et al., 2014) and Golden Gate Assembly (Engler et al., 2008) into one integrated workflow. The targeted assembly step consisted of combining appropriate libraries derived from carrier plasmids (pCP) with the appropriate expression plasmids (pEX) for assembly of Golden Gate to achieve efficient transformants with threefold increased lyocopene titers. The study exhibited efficient assembly of multigene pathways with minimal effort.

In a new paradigm in recombinant production, a synthetic consortium of 4-strain polyculture of *E. coli* was developed to produce 9.5 +0.6 mg/L of callistephin, the red pigment in strawberries (Jones et al., 2017). The polyculture platform overexpressed 15 heterologous enzymes, enabling *de novo* production of anthocyanins from glucose. The study exemplified the potential of polycultures in metabolic engineering to express complex biosynthetic pathways in non-natural hosts. Another study by Levisson et al. (2018) also attempted engineering *Saccharomyces cerevisiae* for the production of pelargonidin 3-O-glucoside, an anthocyanin by introducing the biosynthetic genes from *A. thaliana* and *Gerbera hybrida*. Though the yield obtained (0.01 μmol/g CDW) was low, the work highlighted the potential bottlenecks such as enzyme kinetics, specificity, anthocyanin export which if circumvented could provide a future framework for sustainable production of anthocyanins.

Another promising technique, i.e., Multiplex Automated Genome Engineering (MAGE) strategy can serve as a complementary tool to *de novo* genome synthesis, enabling efficient *in vivo* tuning of genomes for specific applications. This method allows the creation of combinatorial genomic diversity by targeting multiple locations on the chromosome. Wang et al. (2009) automated this technology with the construction of prototype devices and applied it to increase the production of lycopene in *E. coli*. The simultaneous modification of 24 genetic components in the 1-deoxy-D-xylulose-5-phosphate (DXP) pathway resulted in the creation of 4.3 billion genomic variants per day, which led to the isolation of variants with lycopene titers increased more than fivefold.

Another interesting approach that has garnered attention for engineering secondary metabolism is “Multivariate Modular Metabolic Engineering” (MMME), which attempts to reduce regulatory complexities by grouping multiple genes into modules. The various modular engineering approaches for the overproduction of carotenoids have been comprehensively reviewed by Li C. et al. (2020). In context to optimizing carotenoid production using MMME, the biosynthesis pathway is divided into four distinct steps: central carbon module, cofactor module, isoprene supplement module, and carotenoid biosynthesis module. The redirection of carbon flux toward the production of carotenoids while minimizing by-product formation remains the focus of regulating the above four modules. Apart from the selection and engineering of key enzymes, increasing carotenoid storage and membrane-localized expression of enzymes are effective strategies that have been used to achieve better yields (Li C. et al., 2020; Wang et al., 2021). In the context of increasing tolerance of microbial producers to lipophilic carotenoids, the study of Ma et al. (2019) offered valuable insights. The lipid engineering was combined with a systematic metabolic approach to develop an engineered oleaginous biorefinery platform in *S. cerevisiae* which enabled overproduction of lycopene. To increase lycopene accumulation in the non-oleaginous *S. cerevisiae* host, key genes related to fatty acid synthesis and triacylglycerol (TAG) production were overexpressed. This was followed by overexpression of fatty acid desaturase (OLE1) to control TAG fatty acyl composition and regulation of droplet size by deleting seipin (FLD1). The overexpression of key genes, engineered TAG composition and regulated LD size resulted in a 25% increase in lycopene yield reaching 70.5 mg lycopene/g CDW.

In a novel strategy to control the metabolic flux in complex metabolic pathways, Kang et al. (2019) engineered modular enzyme assemblies to develop enhanced cascade catalysis for pigment production. The study successfully demonstrated the effectiveness of scaffold-free enzyme assemblies in both engineered *E. coli* and *S. cerevisiae.* Briefly, the two critical enzymes, i.e., IDI and geranylgeranyl diphosphate synthase (CrtE) were assembled together with help of short peptide tags (RIAD and RIDD). This led to the formation of a pathway node that prevented indirect diffusion and improved production efficiency. The resultant metabolic control caused a 58% increase in the production of lycopene in *S. cerevisiae* cells, reaching 2.3 g/L. Similarly, 5.7-fold higher carotenoid production was achieved in engineered *E. coli.*

In another combined approach of metabolic engineering and mutagenesis, the production of 217.9 mg/L astaxanthin was achieved by engineered *S. cerevisiae* without the addition of inducers (Jin et al., 2018). The heterologous expression of two key enzymes in astaxanthin production, i.e., β-carotene hydroxylase (CrtZ) and β-carotene ketolase (CrtW) was achieved in *S. cerevisiae*, which was followed by ARTP mutagenesis to achieve the highest yield in yeast along with uncovering of new molecular targets for enhancing the isoprenoid production.

Apart from conventional model organisms like *E. coli* and *S. cerevisiae*, the oleaginous yeast *Yarrowia lipolytica* with its GRAS status has also served as an important industrial host for the expression of heterologous genes. Hence, various studies (Celińska et al., 2017; Gao et al., 2017; Larroude et al., 2018) have attempted engineering of this cell factory for pigment production. The construction of 11 β-carotene synthetic genes with iterative integration of multiple copies of those genes enabled the production of 4 g/L β-carotene in engineered *Y. lipolytica*. As compared to the commonly used industrial producer, i.e., *B. trispora* the achieved titer was 1.4-fold higher. Further, the lipid droplets could serve as storage sink for synthesized carotenoids, preventing cytotoxicity to cells (Gao et al., 2017). In another study by Larroude et al. (2018), usage of the Golden Gate DNA assembly toolbox and promoter shuffling helped to identify the best promoter-gene pairs for β-carotene production in engineered *Y. lipolytica.* Combining the synthetic biology approach and fed-batch fermentation optimizations, the titer of 6.5 g/L was achieved for β-carotene with concomitant production of lipids (42.6 g/L).

Overall, all the recent works support the potential of engineering microbial cell factories to overcome the limitations exhibited by native pigment producers. Though many studies have reported improved production at a laboratory scale, the use of engineered microbes for pigment production in an integrated biorefinery system, especially by waste valorization, remains a challenge. Nonetheless, the pigments produced by the biotechnological route are promising for a plethora of applications which are discussed subsequently.

## Multifaceted Applications of Microbial Color Palettes

**FIGURE 2:**
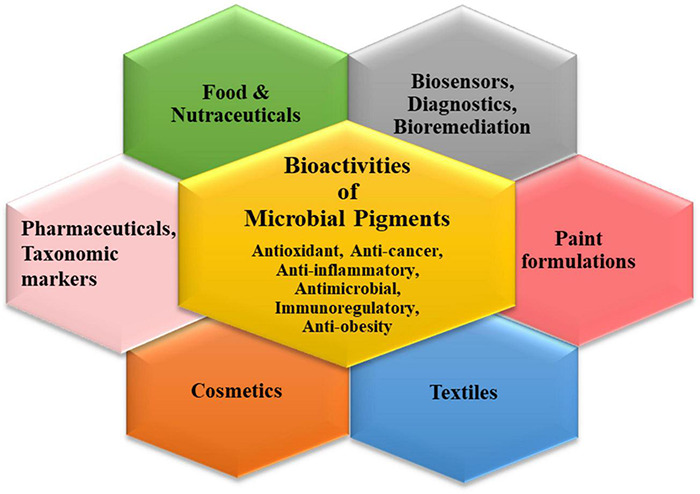
Schematic representation for various applications of microbial pigments.

The plentiful pigments produced by the microbial world have widespread applications in different industrial sectors (Narsing Rao et al., 2017; Chatragadda and Dufossé, 2021; Orlandi et al., 2021; Rana et al., 2021). The characteristic bioactivities of these secondary metabolites endow them with peculiar functional attributes which can be exploited for tailored applications *via* biotechnological tools (Figure 2). Though pigments have been used in cosmetics, bio-indicators, sensors, diagnostic devices, nano-optics, paints, plastics, etc., their indispensable usage in the three main commercial sectors is discussed elaborately.

### Pigments in the Pharmaceutical Industry

The current pandemic of SARS-CoV-2, the emergence of microbial superbugs and alarmingly increasing multidrug-resistance infections have put a high impetus on biomedical research to find putative compounds with anti-pathogenic activity. In this context, microbial pigments have garnered high attention for their pharmacological activities. The majority of the microbial pigments viz. prodigiosin, carotenoids, violacein, flavins, melanins, and quinones have been reported to exhibit antimicrobial, antiproliferative, antioxidant, antiviral, and many more bioactive properties (Narsing Rao et al., 2017; Venil et al., 2020a; Orlandi et al., 2021). Lately, microbial pigments sourced from marine ecosystems have captivated great pharmacological interest due to their therapeutic potential (Torregrosa-Crespo et al., 2018; Chatragadda and Dufossé, 2021; Nawaz et al., 2021). Recently, an astaxanthin pigment purified from a marine endophytic bacteria *Pontibacter korlensis* AG6 was shown to exhibit a cytotoxic effect on the human breast cancer cell line (MCF-7) along with significant antibacterial and anti-oxidant properties (Pachaiyappan et al., 2021).

In another study (Abdelfattah et al., 2019), the prodigiosins produced from an actinomycete, isolated from a marine sponge exhibited gastroprotective effects equivalent to the standard anti-gastric ulcer agent omeprazole. Their antioxidant and anti-inflammatory mechanisms made them potent pharmaceutical targets for preventing gastric damage. Similarly, the prodigiosin produced by two marine isolates, i.e., *Streptomyces* sp. and *Zooshikella* sp. exhibited effective antibacterial activity against *S. aureus* and other human pathogenic strains (Ramesh et al., 2020). The C50 carotenoid bacterioruberin produced by haloalkaliphilic archaeon *Natrialba* sp. M6 demonstrated caspase-mediated apoptotic anticancer properties as well as antiviral potency against hepatitis B and C virus (Hegazy et al., 2020).

Amidst the fungal pigments, though many species such as *Penicillium, Aspergillus, Talaromyces*, and *Fusarium* have been reported to exhibit anti-microbial and anti-oxidant activities (Lagashetti et al., 2019; Morales-Oyervides et al., 2020a), the *Monascus* sp. pigments have been most widely studied as more than 50 pigments with different colorations have been identified from it. The *Monascus* sp. pigments have been reported to exhibit anti-inflammatory, anti-diabetic, anti-obesity, anticancer, and antimicrobial properties making them promising biomolecules for pharmaceuticals (Vendruscolo et al., 2016; Kim and Ku, 2018; He et al., 2021).

### Pigments in the Food Industry

The foods with colorful attractive appearances along with nutritional and health-promoting properties for increased consumer acceptance, are always in demand in the food industry. Therefore, the natural and safe food colorants as an alternative to synthetic ones such as vat green, tartrazine, reactive blue, and sunset yellow are highly sought after in food industries (Rana et al., 2021). The market potential of microbial carotenoids is expanding rapidly in the food industry due to the safe enhancement of food organoleptic properties along with the addition of nutraceutical attributes such as elimination of free radicals, protecting against aging, and many other diseases (Mussagy et al., 2021; Rana et al., 2021). However, the use of microbial pigments as colorants is also challenging as these might fade or lose shelf life due to their higher sensitivity to environmental conditions like light, pH, temperature, oxygen, heat, etc. Therefore, packaging approaches like microencapsulations, nano-emulsions, and nanoformulations are envisaged as effective strategies to improve their stability in food matrices (Sen et al., 2019; Jurić et al., 2020). The encapsulation of violet pigment produced by *C. violaceum* in Gum Arabic followed by spray-drying was shown to be a safe and stable natural colorant for jelly and yogurt (Venil et al., 2015).

Many of the microbial pigments such as β-carotene, lycopene from *B. trispora*, astaxanthin from various bacteria, algae, and *X. dendrorhous*, Arpink red from *Penicillium oxalicum*, pigments from *Monascus* sp., riboflavin from *Ashbya gossypii, Debaryomyces globosus, Eremothecium ashbyii*, and *Candida guilliermondii* are already being used commercially in various foods and beverages (Sen et al., 2019; Venil et al., 2020a; Meruvu and Dos Santos, 2021). Though, many other microbial strains especially fungal sp. exhibit potential as food colorants but requires to be subjected to extensive toxicity and quality tests before being approved by regulatory food authorities for commercial usage (Dufossé, 2018a; Chatragadda and Dufossé, 2021; Poorniammal et al., 2021). The market potential of anthraquinone and azaphilone-producing strains such as *Talaromyces* sp., *Penicillium* sp. along with toxin-free production of *Monascus* sp. pigments for food-grade colorations has been highlighted by various studies (Dufossé, 2018b; He et al., 2021; Poorniammal et al., 2021). Apart from human consumption, the carotenoid pigments have high demand as food additives for animals or aquatic organisms (Meléndez-Martínez et al., 2020; Pereira da Costa and Campos Miranda-Filho, 2020). The carotenoid producing *R. faecalis* PA2 was cultured in domestic wastewater and shown to exhibit price competitiveness for use as animal feed additive (Saejung and Ampornpat, 2019).

### Pigments in the Textile Industry

More than 200,000 tons of dye effluents are generated annually, which cause persistent pollution and pose health hazards (Narsing Rao et al., 2017). To counter the perilous environmental challenges, health risks, allergenicity poised by synthetic dyes, the use of microbial pigments as safe colorants have generated a fervent interest in the textile sector. Further, the added advantage of incorporating antimicrobial properties to develop protective clothing will help to reduce hospital-acquired infections as well as increase consumer acceptance, especially in the post-pandemic era. Recently, the antimicrobial pigment prodigiosin produced from *S. marcescens* SB08 was shown to exhibit effective and stable dyeing efficiency for cotton as well as silk fabrics. The antimicrobial effect was also demonstrated in the pigment soaked textile yarn materials (Venil et al., 2021). Similarly, another study reported formulation of nano-suspension dyeing, based on prodigiosin production by *S. marcescens.* The work testified to the cost-effectiveness of the green process of dyeing acrylic fabric, which also endowed it with antibacterial functionality along with rich color (Ren et al., 2021).

Amidst the fungal genera, the biopigments produced by *Monascus, Aspergillus, Talaromyces, Fusarium, Penicillium, Trichoderma, Scytalidium, Chlorociboria, Curvularia, Cordyceps*, *Alternaria*, and *Phymatotrichum* have been reported for dyeing versatile fabrics (Venil et al., 2020b; He et al., 2021; Meruvu and Dos Santos, 2021). Nonetheless, the evaluation of the toxicity of these fungal metabolites remains a concern before their application. In a study by Hernández et al. (2019) the pigments produced by *Penicillium murcianum* and *Talaromyces australis* were found to be safe for dyeing after the cytotoxicity of lixiviates of dyed wool fabrics as well as fungal pigments was checked on mammalian cell lines NIH/3T3 and HEK293. Apart from native microbial producers, genetic engineering strategies can also boost pigment usage in textile industries. In this context of producing sustainable dyes, a promising study by Ghiffary et al. (2021), demonstrated the production of indigoidine by creating an engineered *C. glutamicum*. The metabolically engineered *C. glutamicum* strain produced the highest titer reported so far, i.e., 49.30 g/L indigoidine from fed-batch fermentation. In comparison to synthetically produced blue dyes, the produced pigment was shown to exhibit similar color properties and fastness on cotton fabrics.

## Conclusion and Future Perspectives

The depleting fossil reserves, increasing population, waste generation, carbon footprint, and resulting detrimental environmental consequences are driving the shift from linear to closed-loop bioeconomy. In this context, the development of microbial cell factories for pigment production using agro-industrial residues seems a rational approach. Though the utilization of these cheap and renewable feedstocks is enticing, the real-time scaled up production and downstream processing of microbial pigments has many challenges. The development of simpler downstream approaches along with enhanced stability and shelf life of microbial pigments by entrapment strategies like nano-emulsions, encapsulation will help in paving the road toward market sustainability in competition with synthetic commodities. Overall, fostering these innovative and green bioprocesses to commercial scale will require extensive research efforts from inter-disciplinary pursuits and the amalgamation of supportive regulatory policies.

Various biotechnological tools viz. metabolic engineering, mutagenesis, homologous or heterologous overexpression, and other genetic manipulations will play a vital role in strain improvement for high-titer pigment production utilizing residues. Nonetheless, to counter the concerns in using native or engineered microbial pigments for commercial usage, requires passing through quality and toxicological tests to achieve regulatory bodies approval. In addition to advanced understanding of complex intertwined biosynthetic metabolic pathways and development of engineered microbial platforms, the biological and physicochemical fermentation conditions need to be optimized for high product yield with minimal contaminants or by-products. In summary, the realization of trash to treasure approach will depend on a functionally integrated bioprocess developed by technological advancements and optimizations for each contributing parameter, i.e., from pretreatment of feedstocks, fermentation technologies, tailored microbial strains, effective extraction, and purification protocols to stable product formulation.

## Author Contributions

KP and JG: conceptualization. JG, MW, WP, and NJ: writing—original draft preparation and literature search. JG, KP, and LD: writing—review and editing. KP and LD: supervision. All authors have read and agreed to the published version of the manuscript.

## Conflict of Interest

The authors declare that the research was conducted in the absence of any commercial or financial relationships that could be construed as a potential conflict of interest.

## Publisher’s Note

All claims expressed in this article are solely those of the authors and do not necessarily represent those of their affiliated organizations, or those of the publisher, the editors and the reviewers. Any product that may be evaluated in this article, or claim that may be made by its manufacturer, is not guaranteed or endorsed by the publisher.
